# A comparative study between mixed-type tumours from human salivary and canine mammary glands

**DOI:** 10.1186/1471-2407-7-218

**Published:** 2007-11-28

**Authors:** Marisa CLS Genelhu, Sérgio V Cardoso, Helenice Gobbi, Geovanni D Cassali

**Affiliations:** 1 Laboratory of Comparative Pathology, Biological Sciences Institute, Federal University of Minas Gerais, Belo Horizonte, Minas Gerais, Brazil; 2 Department of Pathology and Forensic Medicine, School of Medicine, Federal University of Minas Gerais, Belo Horizonte, Minas Gerais, Brazil; 3 Immunology Research Laboratory, Vale do Rio Doce University, Governador Valadares, Minas Gerais, Brazil; 4 Department of Dentistry, State University of Montes Claros, and Oral Pathology Laboratory, School of Dentistry, Federal University of Uberlândia, Minas Gerais, Brazil

## Abstract

**Background:**

In comparative pathology, canine mammary tumours have special interest because of their similarities with human breast cancer. Mixed tumours are uncommon lesions in the human breast, but they are found most frequently in the mammary gland of the female dogs and in the human salivary glands. The aim of the study was to compare clinical, morphological and immunohistochemical features of human salivary and canine mammary gland mixed tumours, in order to evaluate the latter as an experimental model for salivary gland tumours.

**Methods:**

Ten examples of each mixed tumour type (human pleomorphic adenoma and carcinomas ex-pleomorphic adenomas and canine mixed tumour and metaplastic carcinoma) were evaluated. First, clinical and morphologic aspects of benign and malignant variants were compared between the species. Then, streptavidin-biotin-peroxidase immunohistochemistry was performed to detect the expression of cytokeratins, vimentin, p63 protein, estrogen receptor, β-catenin, and E-cadherin.

**Results:**

After standardization, similar age and site distributions were observed in human and canine tumours. Histological similarities were identified in the comparison of the benign lesions as well. Metaplastic carcinomas also resembled general aspects of carcinomas ex-pleomorphic adenomas in morphological evaluation. Additionally, immunohistochemical staining further presented similar antigenic expression between lesions.

**Conclusion:**

There are many similar features between human salivary and canine mammary gland mixed tumours. This observation is of great relevance for those interested in the study and management of salivary gland tumours, since canine lesions may constitute useful comparative models for their investigations.

## Background

Animal models have been widely used to investigate several forms of human neoplasias. Because of centuries of coexistence with humans in the same environment, dogs are of particular interest as they provide important evolutionary information. In addition, both species show great genotypic similarities [1]. Thus, spontaneous tumours of canine mammary glands have been proposed as comparative models for the study of human breast cancer, since these lesions share epidemiological, clinical, behavioural and antigenic features [2-5].

There is also a well-known relationship between the incidence of human mammary and salivary glands tumours [6-9]. Morphological similarities have been described between certain tumours of salivary glands and breast neoplasias such as those existing between polymorphous low-grade adenocarcinoma and invasive lobular carcinoma [10], between acinic cell carcinoma and invasive secretory carcinoma [11], and between epithelial-myoepithelial carcinoma and adenomyoepithelioma [12]. Ductal carcinomas [13,14], adenoid cystic carcinomas and mixed tumours with similar patterns may be found in both organs [15,16].

Mixed tumours are unusual lesions in the human breast [17], but they are frequent in both human salivary and canine mammary glands [18-20]. In a comparative evaluation of the available literature, pleomorphic adenoma (PA) and its malignant counterpart, the carcinomas ex-pleomorphic adenomas (Ca ex-PA) have several interesting similarities to benign mixed tumours (MT) and to metaplastic carcinomas (MC) of canine mammary glands. First, all of them are derived from exocrine glands, which depict similar tissue architecture. Next, with few variations, both are microscopically characterized by a mixture of ductal and myoepithelial elements intermingling an apparently mesenchymal stroma of variable constitution [18-20]. In addition, malignant transformation is acknowledged for both for human PA and canine MT, particularly in lesions with long evolution and frequent recurrences [20-25]. In spite of these similar aspects, to the best of our knowledge no specific comparative investigation between human salivary and canine mammary glands tumours is available.

Thus, the present work aimed to perform objective morphological microscopic comparison between mixed tumours derived from human salivary and canine mammary glands, as well as to evaluate the immunohistochemical expression of some relevant antigens in order to characterize these two types of neoplastic alterations.

## Methods

### Samples

Ten samples of PA and 10 of Ca ex-PA were obtained from the Department of Pathology of School of Medicine, Federal University of Minas Gerais (UFMG, Belo Horizonte, Minas Gerais, Brazil), A. C. Camargo Cancer Hospital (São Paulo, São Paulo, Brazil), and the National Cancer Institute (Rio de Janeiro, Rio de Janeiro, Brazil). Ten samples of MT and 10 of MC of mammary glands of dogs without defined breed were obtained from the records of the Laboratory of Comparative Pathology, Biological Sciences Institute, UFMG. Ca ex-PA diagnosis was restricted to cases with clinical features (such us a previous benign tumour excised from a site in which recurrent malignant tumour), and/or histological evidence of arising in or from a benign lesion (identification of at least a focus benign tumour) [18]. The clinical analyses of the tumours studied are summarized in Tables 1, 2, 3, 4. The malignant components of Ca ex-PA were further identified and subtyped according to World Health Organization (WHO) and Armed Forces Institute of Pathology (AFIP) criteria [18,26] and are summarized in Table 2.

**Table 1:** Clinical characterization of human salivary glands pleomorphic adenomas. (M – male; F – female)

**Case**	**Age**	**Gender**	**Gland**
1	30	M	minor (lip)
2	35	F	parotid
3	42	M	parotid
4	53	F	parotid
5	29	F	parotid
6	51	F	parotid
7	32	F	parotid
8	39	F	submandibular
9	58	M	parotid
10	27	M	parotid

**Table 2:** Clinical and histological subtypes of human salivary glands carcinomas ex-pleomorphic adenomas (M – male; F – female)

**Case**	**Age (years)**	**Gender**	**Gland**	**Histological Subtype**
1	33	F	minor (palate)	adenocarcinoma NOS
2	22	F	submandibular	adenocarcinoma NOS
3	71	M	parotid	undifferentiated carcinoma
4	53	F	minor (palate)	myoepithelial carcinoma
5	43	M	Parotid	myoepithelial carcinoma
6	02	M	parotid	myoepithelial carcinoma
7	65	F	parotid	adenoid cystic carcinoma (solid)
8	70	M	minor (palate)	adenoid cystic carcinoma (tubular)
9	92	F	submandibular	polymorphous low-grade adenocarcinoma
10	66	M	parotid	mucoepidermoid carcinoma

**Table 3:** Clinical characterization of benign mixed tumours of canine mammaryglands

**Case**	**Age**	**Gender**	**Localization**
1	5	F	Inguinal
2	7	F	Thoracic-cranial
3	9	F	Abdominal-caudal
4	4	F	Abdominal-caudal
5	6	F	Thoracic-cranial
6	3	F	Inguinal
7	7	F	Abdominal-caudal
8	5	F	Inguinal
9	7	F	Inguinal
10	8	F	Inguinal

**Table 4:** Clinical characterization of malignant mixed tumours of canine mammary glands

**Case**	**Age**	**Gender**	**Localization**
1	7	F	Thoracic-cranial
2	13	F	Thoracic-caudal
3	5	F	Abdominal-cranial
4	8	F	Inguinal
5	9	F	Thoracic-caudal
6	8	F	Inguinal
7	9	F	Inguinal
8	9	F	Thoracic-cranial
9	6	F	Thoracic-cranial
10	4	F	Abdominal-cranial

All samples were formalin-fixed, paraffin-embedded, and new histological sections were independently reviewed by two experienced observers to confirm the diagnosis. Clinical and demographic data (age, gender, affected salivary or mammary gland) from affected individuals were retrieved from medical and veterinary charts.

### Immunohistochemistry

To further compare tumours, immunohistochemical assays were carried out to detect antigens related to cellular differentiation (cytokeratins, vimentin, and p63), adhesion (E-cadherin and β-catenin), and hormonal status (estrogen receptor), which have been shown to be relevant for the study of human salivary and breast cancer [27-36]. Streptavidin-biotin-peroxidase technique was used, employing the antibodies described in Table 5. Briefly, 3 μm thick histological sections were deparaffinised in xilol and dehydrated in decreasing alcohol concentrations. Next, they were submitted to antigenic retrieval (Target Retrieval Solution, pH 6.0, DakoCytomation, Carpinteria, USA) and endogenous peroxidase blocking (3% hydrogen peroxide in methanol). After incubation with primary antibodies (Table 5) and amplification (Ultra Vision Large Volume Detection System, Lab Vision, Fremont, USA), the reaction was revealed with diaminobenzidine as chromogen and Mayer haematoxylin as contrast. As positive controls, sections of normal human salivary and mammary glands with previously recognized positivity for the antigens studied were used. Substitution of primary antibody for normal human serum constituted the negative control.

**Table 5:** Primary antibodies, resources and dilutions used in immunohistochemical assays

**Antibody**	**Clone**	**Resource**	**Dilution**
Anti-pan-cytokeratin	NCL-AE1/AE3	Novocastra	1:100
Anti-vimentin	V9	DAKO	1:50
Anti-p63	4A4	Santa Cruz	1:100
Anti-β-catenin	E-5	Santa Cruz	1:400
Anti-E-cadherin	4A2C7	Zymed	1:40
Anti-estrogen receptor	CC4–5	Novocastra	1:50

Finally, morphological analysis of staining was performed, and then a semiquantitative protocol was employed to segregate the cases. For this latter purpose the entire available tumoural tissue in the sections was evaluated. Next, it was determined whether the relative number of positive neoplastic cells was superior ("positive cases") or inferior ("negative cases") to 5% (for the analysis of p63 and estrogen receptor) [37,38] or 10% (cytokeratins, vimentin, E-cadherin, β-catenin) [29,32] from all of the neoplastic cells in the histological sections evaluated.

### Statistical analysis

Frequency of positive immunostaining between the four groups of lesions was evaluated by Fisher's exact test with values of p < 0.05 considered statistically significant. Probability of α-error inferior to 5% was confirmed to be significant.

## Results

In both species it was observed that the benign tumours occur in the younger individuals' group, while the malignant tumours are more frequent in older individuals' group. A slight predominance of female patients (six cases) was observed for PA, while a homogeneous distribution was observed among those patients with Ca ex-PA. In dogs, all lesions affected females.

**Figure 1:**
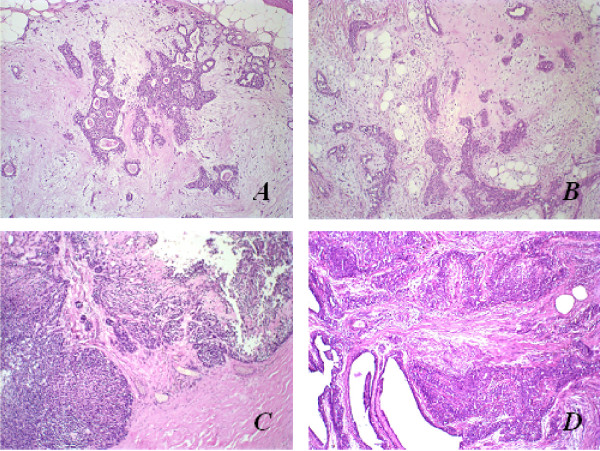
Histopathological aspects of benign and malignant mixed tumours of human salivary and canine mammary glands by haematoxylin and eosin (HE) stain; original magnification, 10×. **(A) **Pleomorphic adenoma in human salivary gland; **(B) **Mixed tumour in canine mammary gland; **(C) **Carcinoma ex-pleomorphic adenoma in human salivary gland; **(D) **Metaplastic carcinoma in canine mammary gland.

Histomorphological comparative illustrations are exemplified in Figure 1. In general, both benign tumours presented formation of ductal structures and also cells with myoepithelial features, arranged in solid aggregations, cords, nests, or even isolated, but irregularly dispersed in a predominantly myxoid or myxo-chondroid matrix. MC were observed to be histomorphologically similar to the adenocarcinoma NOS (not-otherwise specified) or to undifferentiated carcinoma-type Ca ex-PA, since both were infiltrative lesions with malignant degeneration areas, characterized by cell pleomorphism (ovoid to polyhedral cells with clear to hyaline cytoplasm), and hyperchromatic or vesiculated nuclei with conspicuous nucleoli.

Immunohistochemical assays displayed positive cytoplasmic localization of cytokeratins in all neoplastic cells from all lesions. Vimentin was identified in the cytoplasm of non-luminal cells of ductal formations, in plasmacytoid and spindle cells of PA and canine MT, in all MC cells, and was identified diffusely in Ca ex-PA with myoepithelial differentiation (those which the malignant component was described as myoepithelial, adenoid cystic, and polymorphous low-grade adenocarcinomas).

**Figure 2:**
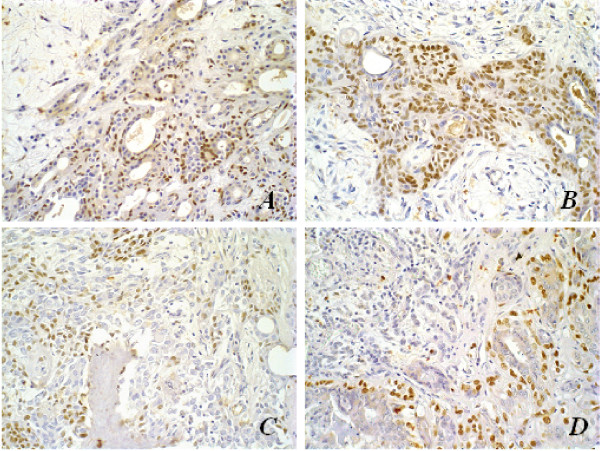
Immunohistochemical aspects of p63 antigen stain; original magnification, 40×. **(A) **Pleomorphic adenoma in human salivary gland; **(B) **Mixed tumour in canine mammary tumour; **(C) **Carcinoma ex-pleomorphic adenoma in human salivary gland; **(D) **Metaplastic carcinoma in canine mammary gland. Note myoepithelial p63-negative cells in malignant tumours (arrows).

All PA presented positive p63 nuclear immunolocalization in neoplastic luminal, plasmacytoid and spindle cells, while p63 was found in only five samples of Ca ex-PA (two samples with diagnosis of myoepithelial carcinoma, two with adenoid cystic carcinomas, and one with undifferentiated carcinoma). All canine MT and MC depicted positive p63 expression, in a similar fashion to that seen in PA, while the malignant lesion had less positive cells and these presented less intense reaction (Figure 2). The decrease in immunopositivity frequency in Ca ex-PA was significantly different regarding all the other groups (p < 0.05).

**Figure 3:**
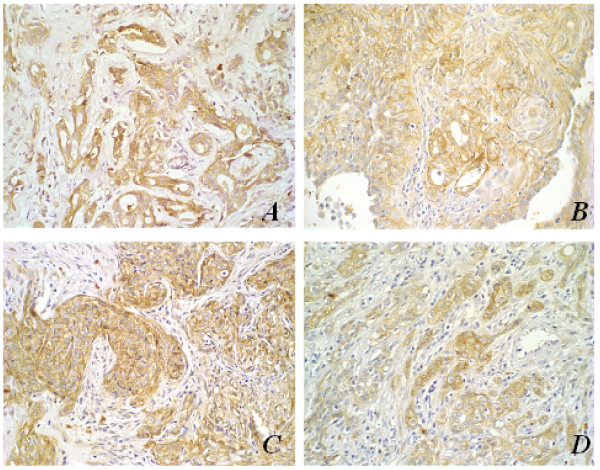
Immunohistochemical aspects of β-catenin antigen stain; original magnification, 40×. **(A) **Pleomorphic adenoma in human salivary gland with membrane and cytoplasmic β-catenin stain; **(B) **Mixed tumour in canine mammary tumour with membrane and cytoplasmic β-catenin stain; **(C) **Carcinoma ex-pleomorphic adenoma in human salivary gland showing β-catenin nuclear stain (arrows); **(D) **Metaplastic carcinoma in canine mammary gland showing β-catenin nuclear stain (arrows).

β-catenin expressions in neoplastic epithelial cells of both PA of human salivary gland and MT of canine mammary glands have shown to be similar in location, to the expression in normal glandular parenchyma (membrane and/or cytoplasmic). Membrane and cytoplasmic β-catenin immunolocalization was especially frequent in cells of ductal formations in these benign tumours. In malignant lesions, nuclear expression of this protein was also identified (Figure 3). No β-catenin expression was observed in highly atypical areas of malignant tumours of both species or in those with rich myxo-chondroid stroma.

**Figure 4:**
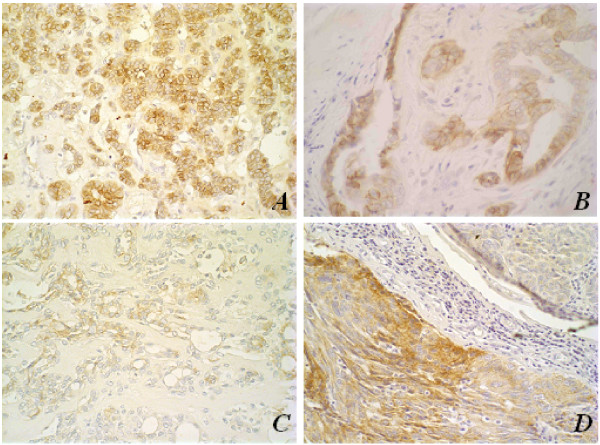
Immunohistochemical aspects of E-cadherin membrane antigen stain; original magnification, 40×.**(A) **Pleomorphic adenoma in human salivary gland with membrane E-cadherin stain; **(B) **Mixed tumour in canine mammary gland with membrane E-cadherin stain; **(C) **Carcinoma ex-pleomorphic adenoma in human salivary gland with evident loss of membrane E-cadherin marker; **(D) **Metaplastic carcinoma in canine mammary gland with evident loss of E-cadherin membrane marker. Note the loss of expression in malignant tumours.

Analysis of E-cadherin expression has shown that benign tumours of both species presented a similar linear membrane expression to that of normal gland parenchyma. Malignant neoplasias depicted less intense expression, which was also related to poor cell differentiation (Figure 4).

Estrogen receptor expression was not identified in tumours from the human salivary gland, while all the canine mammary tumours presented immunoreactivity for this marker (p < 0.05).

## Discussion

The study of animal tumours may provide data to improve the understanding of similar lesions in humans, as well as tumour aetiology and development [39]. To date, there is not a universally accepted animal model for neoplastic pathology investigations. Transgenic mice, for instance, have received criticism as some of these animals are refractory to the development of certain types of tumours despite presentation of the same genetic lesion [40]. Animal cell culture is also an imperfect comparative model, as many of the events associated with carcinogenesis variably depend on a host [41]. One of the great advantages of canine breast model is that tumours are spontaneous in this organ. As its clinical evolution is natural, genetic and morphophysiological aspects may be better compared with some aspects of the human species [42].

Dogs represent a remarkable incidence of neoplasia development, usually associated with environmental exposure to important carcinogens for humans [1,43]. Tumours effecting the mammary glands, especially in females, are among the most frequent tumours observed in dogs. Finally, mixed tumours are one of the most frequent neoplasias in dogs [19] with remarkable features in common with human salivary gland tumours, justifying the investigation of other possible similarities between lesions of these two species.

The present study has confirmed that canine mammary gland MT and MC share some clinical characteristics with human salivary gland PA and Ca ex-PA, including age of emergence and several histopathological aspects. We have also demonstrated that commercially available antibodies for the study of human neoplasias are functional to detect antigenic expression in canine lesions. Moreover, similar antigenic expressions (for cytokeratins, vimentin, β-catenin, and E-cadherin) were identified between the lesions, suggesting common pathogenetic mechanisms in the histogenesis of these tumours.

Vimentin and the pool of cytokeratins detected by the monoclonal antibody AE1/AE3 depicted parallel immunolocalization pattern, confirming the utility of this antigen to biologically characterize tumoural components.

p63 immunolocalization was restricted to cells with myoepithelial morphological features in PA, and to lesions of myoepithelial differentiation in Ca ex-PA, albeit the less intense staining in the latter suggests some loss of differentiation [44]. Similar observations were found for canine tumours, corroborating the use of this marker to demonstrate myoepithelial cells in both species. However, the analysis of the antigenic behaviour in tumours of both species is hindered by the fact that p63 possesses two different isoforms (TAp63 and ΔNp63) with opposite functions, being responsible for cell-cycle arrest and cell proliferation, respectively [45]. Clone 4A4, used in our study, recognizes all isoforms. It remains to be further evaluated by future works.

The expression of cell adhesion relating β-catenin and E-cadherin proteins was also similar in both species. In PA and canine MT, β-catenin and E-cadherin presented with predominantly membrane expression. β-catenin expression in MC and Ca ex-PA was either cytoplasmic/nuclear, or only nuclear, suggesting that changes in antigenic location may be related to the induction of gene transcription linked to cell proliferation in malignant tumours [34,35,45]. In addition, the loss of β-catenin and E-cadherin membrane expression may be associated with more aggressive tumour characteristics such as invasiveness and metastasis [46-49].

The most outstanding difference in antigenic expression was related to estrogen receptor. ER immunostaining was observed in all canine lesions, and it was not detected in any human neoplasia evaluated. Several previous studies showed the presence of ER in canine mammary tumours, suggesting this protein participates in lesion formation [50-52]. The lack of immunolocalization in salivary gland tumours has been reported also by others [38,53-55]. One possible explanation would be the very low level expression of this protein [56], with an mRNA transcription being observed, which was shown by the study of Leimola-Virtanen *et al*. [57], or difficulties in recognition of epitopes through immunohistochemistry. In this work, the absence of ER expression in human salivary gland tumours suggests that these lesions not very responsive to estrogen, in contrast to the lesions in dogs, but further studies should be carried out to better define the role this protein in salivary gland tumorigenesis.

## Conclusion

In the present work, some clinical, histopathological and antigenic similarities were confirmed between mixed-type tumours from human salivary and canine mammary glands. These data could suggest a hypothesis of similar histogenesis between these neoplasias. More interestingly, it encourages the use of spontaneous canine mammary gland tumours as animal models to study human salivary gland mixed neoplasias. However, differences were also identified and, therefore, additional studies should be carried out to better define advantages and disadvantages of a comparative assessment between these lesions.

## Competing interests

The author(s) declare that they have no competing interests.

## Authors' contributions

All authors contributed to the writing of the manuscript. All authors read and approved the final manuscript.

## Pre-publication history

The pre-publication history for this paper can be accessed here:


